# Silymarin Induces Expression of Pancreatic Nkx6.1 Transcription Factor and β-Cells Neogenesis in a Pancreatectomy Model

**DOI:** 10.3390/molecules19044654

**Published:** 2014-04-15

**Authors:** Claudia Soto, Luis Raya, Julia Pérez, Imelda González, Salud Pérez

**Affiliations:** 1 Departamento de Sistemas Biológicos, Universidad Autónoma Metropolitana-Xochimilco, México City 04960, Mexico; E-Mails: jperez@correo.xoc.uam.mx (J.P.); ha_des_dark@hotmail.com (I.G.); msperez@correo.xoc.uam.mx (S.P.); 2 Facultad de Medicina, Universidad Nacional Autónoma de México, México City 04510, Mexico; E-Mail: andres.2508@hotmail.com

**Keywords:** insulin, pancreatic regeneration, pancreatic transcription factors

## Abstract

A physio-pathological feature of diabetes mellitus is a significant reduction of β-pancreatic cells. The growth, differentiation and function maintenance of these cells is directed by transcription factors. Nkx6.1 is a key transcription factor for the differentiation, neogenesis and maintenance of β-pancreatic cells. We reported that silymarin restores normal morphology and endocrine function of damaged pancreatic tissue after alloxan-induced diabetes mellitus in rats. The aim of this study was to analyze the effect of silymarin on Nkx6.1 transcription factor expression and its consequence in β cells neogenesis. Sixty male Wistar rats were partially pancreatectomized and divided into twelve groups. Six groups were treated with silymarin (200 mg/Kg p.o) for periods of 3, 7, 14, 21, 42 and 63 days. Additionally, an unpancreatectomized control group was used. Nkx6.1 and insulin gene expression were assessed by RT-PCR assay in total pancreatic RNA. β-Cell neogenesis was determined by immunoperoxidase assay. Silymarin treated group showed an increase of Nkx6.1 and insulin genic expression. In this group, there was an increment of β-cell neogenesis in comparison to pancreatectomized untreated group. Silymarin treatment produced a rise in serum insulin and serum glucose normalization. These results suggest that silymarin may improve the reduction of β pancreatic cells observed in diabetes mellitus.

## 1. Introduction

In diabetes mellitus there is a loss or dysfunction of β-pancreatic cells which are key glucose homeostasis regulators. Type 1 diabetes results from autoimmune destruction of insulin producing β-pancreatic cells, whereas type 2 diabetes involves loss of glucose stimulated insulin secretion and a gradual diminution of β-cell mass [1]. It has been shown that the development, differentiation and functioning of β-pancreatic cells are controlled by transcription factors [2]. The Nkx6.1 homeodomain factor directs the differentiation specifically of the insulin secreting β-cell [3]. Targeted disruption of Nkx6.1 in mice leads to the near absence of β-cells neogenesis, whereas all other islet cell types appear to develop normally [4]. In the developing mouse pancreas, which begins its formation at embryonic day e9.5 [5], Nkx6.1 protein could be detected at embryonic day e10.5, and persisted through e12.5. At e13 Nkx6.1 expression becomes restricted to β-cells [6] and is a necessary component of the signals triggering the major wave of their differentiation and proliferation [7]. By e15.5 it was exclusively detected in insulin-expressing cells and scattered ductal and periductal cells. At e15.5, other pancreatic transcription factors, Pdx-1 and the bHLH, factor neurogenin 3 (Ngn3), a marker of islet cells progenitors, co-expressed with Nkx6.1 expression [8]. Pdx-1 is the key transcription factor for the development, differentiation, maintenance and survival of an adequate mass of healthy β-cells in adults. Pdx-1 is required for maintaining Nkx6.1 expression in differentiated β-cells [9]. Schaffer *et al.* [10] reported that this transcription factor is both necessary and sufficient to specify insulin producing β-cells and maintain an adequate mass of these cells in adults. We previously reported that silymarin restores the normal morphology and endocrine function of damaged pancreatic tissue in alloxan induced diabetic rats [11]. Silymarin is a standardized extract of *Silybum marianum,* composed of the flavolignans silybin, isosilybin A, isosilybin B, silychristin and silydianin, which have proven regenerative properties in several hepatic disorders [12,13]. In this study the effect of silymarin was tested in the gene expression of Nkx6.1 transcription factor and its effect in the neogenesis of pancreatic β-cells after a 60% pancreatectomy in rats, as well as the functionality of the tissue itself.

## 2. Results and Discussion

### 2.1. Nkx6.1 and Insulin Expression

**Figure 1:**
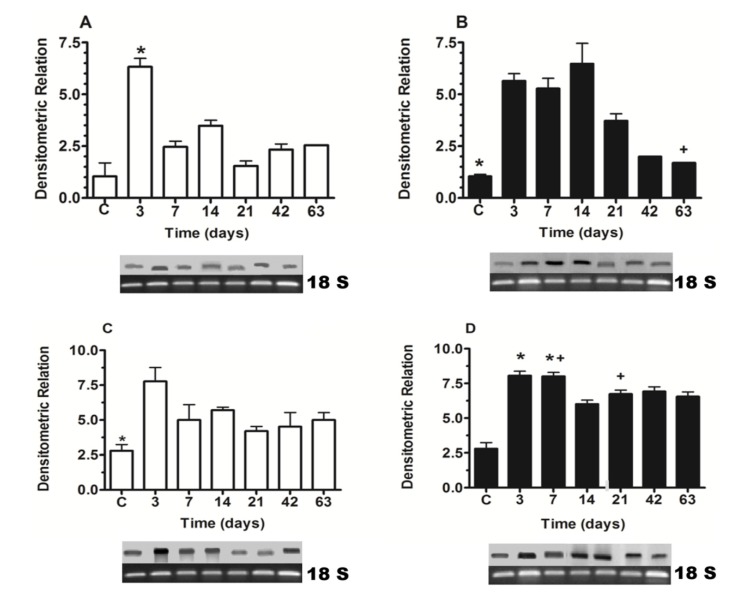
Nkx6.1 (**A**, **B**) and insulin (**C**, **D**) gene expression in pancreatectomized animals. (**A**, **C**) untreated group, (**B**, **D**) silymarin treated group. (**C**) control group. Each bar represents the mean ± S.E.M. * *p* < 0.05 *versus* other times of the same group. + *p* < 0.05 treated *versus* untreated animals at the same times. *n* = 5.

Silymarin induced a significant increment in Nkx6.1 genic expression at 7, 14 and 21 days of treatment, in comparison to same times (days) of untreated pancreatectomized animal groups. After this time, the expression of this factor was decreased in the silymarin treated group, but was maintained at similar values to control group (Figure 1A,B). According to Nkx6.1 expression, the silymarin treatment produced an increase in the insulin genic expression in relation to untreated groups, significantly at 7 and 21 days (Figure 1C,D).

### 2.2. Western Blot of Nkx6.1

**Figure 2:**
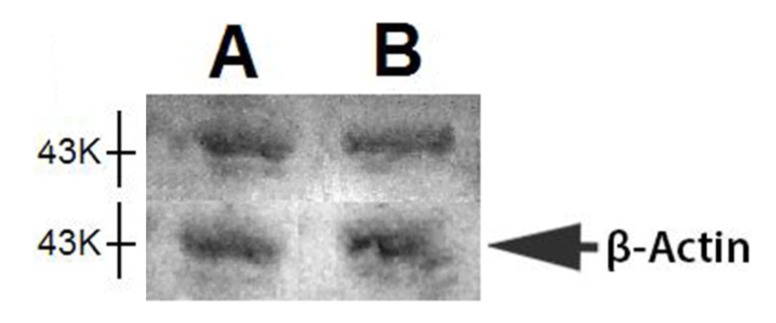
Representative western blot analysis of pancreatic Nkx6.1 transcription factor and β-actin of partial pancreatectomized rats treated with silymarin: (**A**) 21 days; and (**B**) 63 days after pancreatectomy.

Nkx6.1 expression decreased at 42 and 63 days after silymarin treatment (in comparison to other times, Figure 1B). In order to observe a possible decrease in the protein at these times, a western blot analysis of this transcription factor was carried out for pancreatic protein samples of 21 and 63 days of treatment. There was no difference between the results of these times (Figure 2).

### 2.3. Nkx6.1 Immunolabelling

**Figure 3:**
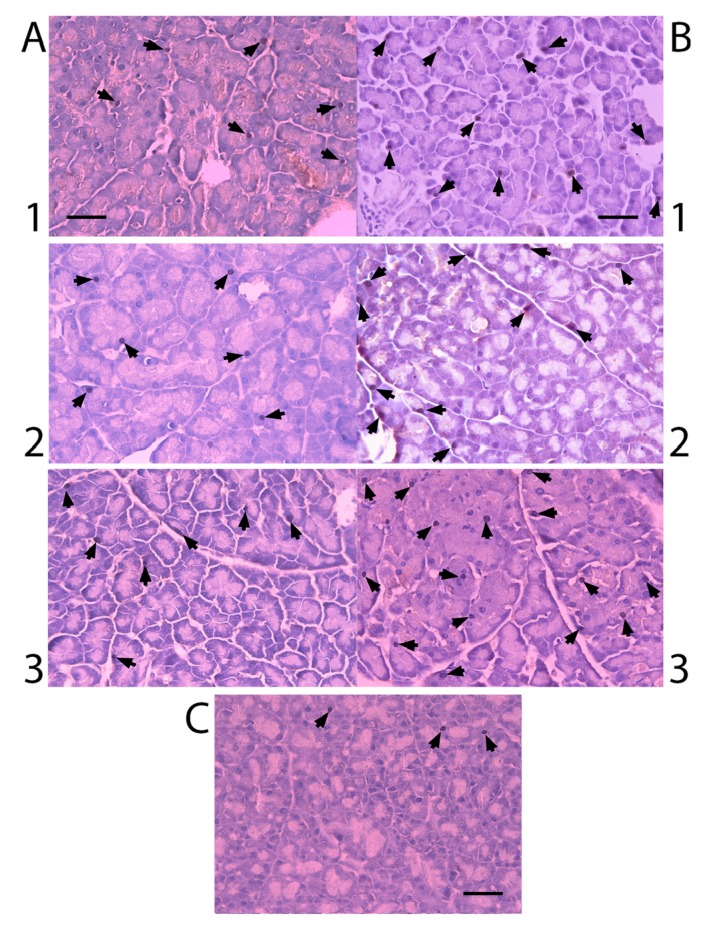
Immunoperoxidase for Nkx6.1 labeling of pancreatectomized rat pancreas (× 400 magnification). Staining (arrows) for Nkx6.1. Scale bar = 25 µm. (**A**) untreated groups; (**B**) silymarin treated groups; (**C**) control group, (1) 7 days after pancreatectomy; (2) 14 days after pancreatectomy; (3) 21 days after pancreatectomy.

**Figure 4:**
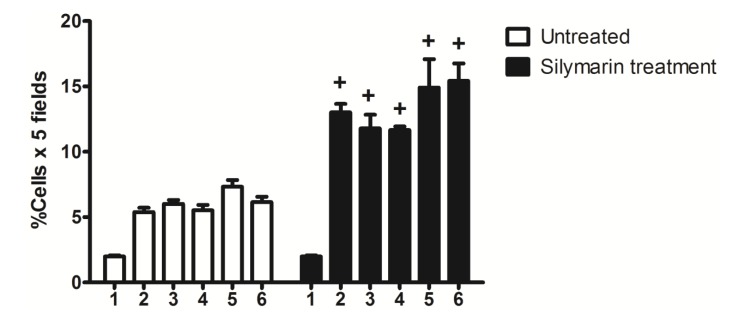
Nkx6.1 immunolabeled cells were counted as percentage of total cells in each field with five fields of each animal pancreatic tissue at different times after pancreatectomy: (1) control; (2) 3 days; (3) 7 days; (4) 14 days; (5) 21 days; (6) 63 days. + *p* < 0.05 treated *versus* untreated animals at the same times. *n* = 5.

All experimental groups presented Nkx6.1 immunoreaction (Figure 3). Cells count of Nkx6.1 immunolabelled cells is shown in Figure 4. Pancreatectomized group presented an increase in these immunolabels in comparison to control group. There was no significant difference of Nkx6.1 immunolabels at different times after the pancreatectomy. Silymarin treated groups showed a greater number of Nkx6.1 immunolabels than untreated groups and there was no significant difference among the groups with different times of treatment after the pancreatectomy.

2.4. β-Cell Neogenesis

**Figure 5:**
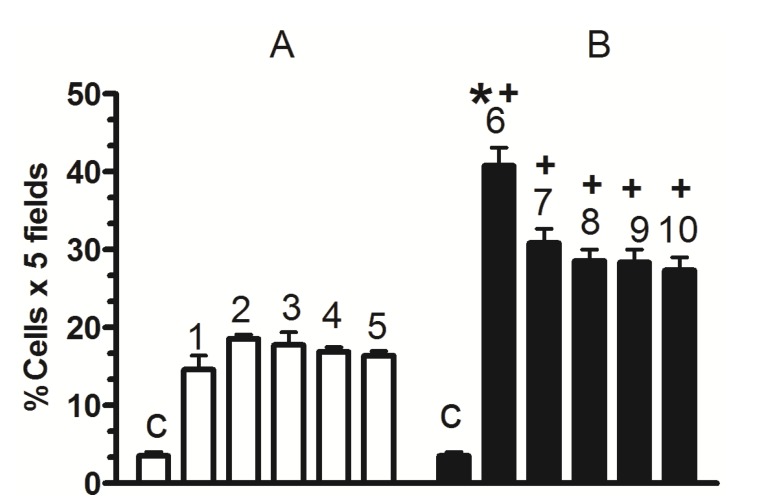
β-cell neogenesis. (**A**) untreated; (**B**) silymarin treated; (**C**) control group, (1) 3 days after pancreatectomy group; (2) 7 days after pancreatectomy group; (3) 14 days after pancreatectomy group; (4) 21 days after pancreatectomy group; (5) 63 days after pancreatectomy group; (6–10) silymarin treated groups with the same days after pancreatectomy for untreated groups. Each bar represents the mean ± S.E.M. * *p* < 0.05 *versus* other times of the same group. + *p* < 0.05 treated *versus* untreated animals at the same times. *n* = 5.

Figure 5 shows that partial pancreatectomy induced a significant increase in β-cell neogenesis (% of cells × 5 fields) that was very similar for all time periods, in comparison to the control group. Silymarin treatment induced a significant increase in β-cell neogenesis (% of cells × 5 fields), that was greater on the 3rd day of treatment. After this time the increase was sustained for all time periods.

**Figure 6:**
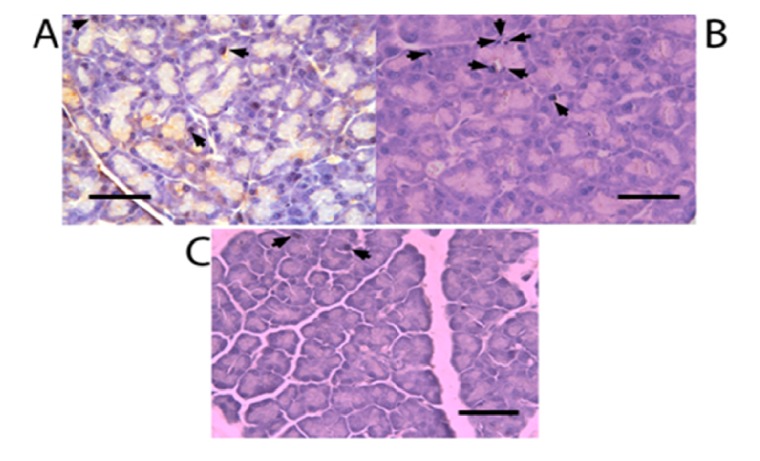
Double immunolabelling for insulin and CK20 of pancreas (arrows) (× 400 magnification). Scale bar = 25 µm. (**A**) untreated group; (**B**) silymarin treated group. Both at 63 days after pancreatectomy; (**C**) control group.

Figure 6 illustrates a duplication in immunolabelling for insulin and CK20 (β-cell neogenesis) for black immunolabels. A greater increase of β-cell neogenesis was observed with the silymarin treatment (Figure 6B) compared to untreated groups (Figure 6A) after the pancreatectomy.

### 2.5. Serum Insulin Levels

The pancreatectomized untreated group showed insulin serum levels under the control values at 3, 14 and 21 days. At 42 and 63 days these levels reach the control. Silymarin treatment maintained hormone serum levels within the control values and significantly augmented at 42 and 63 days compared to other times for this group, as well as those presented at 42 and 63 days for the untreated rats (Table 1).

**Table 1:** Serum insulin levels in experimental groups.

Days after pancreatectomy	Serum insulin (ng/mL) Untreated group	Serum insulin (ng/mL) Silymarin treated group
Control	1.28 ± 0.02	1.28 ± 0.02
3	0.350 ± 0.089	0.777 ± 0.122
7	1.158 ± 0.474	0.487 ± 0.023
14	0.293 ± 0.087	0.505 ± 0.018
21	0.175 ± 0.351	0.782 ± 0.202 ^+^
42	1.224 ± 0.292	2.79 ± 0.342 *^,+^
63	1.123 ± 0.357	3.206 ± 0.272 *^,+^

Serum insulin levels. Each bar represents the mean ± S.E.M. * *p* < 0.05 *versus* other times of the same group; + *p* < 0.05 treated *versus* untreated animals at the same times. *n* = 5.

### 2.6. Serum Glucose Levels

Table 2 shows that serum glucose concentration in pancreatectomized untreated rats was significantly elevated and was maintained above the values of the control group. Contrary to this group, silymarin treated animals showed serum glucose concentration values significantly lower and similar to control group values at 42 days and 63 days of treatment.

**Table 2:** Serum glucose levels in experimental groups.

Days after pancreatectomy	Serum glucose (mM) Untreated group	Serum glucose (mM) Silymarin treated group
Control	5.9 ± 0.004	5.9 ± 0.004
3	9.00 ± 1.575	6.2 ± 0.231
7	7.93 ± 0.493	9.00 ± 0.151
14	9.74 ± 0.788	7.2 ± 0.352 ^+^
21	7.54 ± 0.222	8.02 ± 1.2
42	10.55 ± 0.858	7.25 ± 0.256 ^+^
63	9.34 ± 0.457	6.35 ± 0.252 ^+^

Glucose levels. Each value represents the mean ± S.E.M. + *p* < 0.05 treated *versus* untreated animals at the same times. *n* = 5.

### 2.7. Discussion

We have previously demonstrated that silymarin restores the normal morphology and endocrine function of damaged pancreatic tissue in alloxan-induced diabetic rats. In this study, we showed that silymarin induced an increase of both Nkx6.1 and insulin gene expression as well as β-cell neogenesis in partially pancreatectomized rats (compared to pancreatectomized untreated animals). These results caused an increase in insulin serum levels and a normalization of blood glucose levels comparable to control values. Up until now there have been no reports on these effects of silymarin.

The Nkx6.1 transcription factor plays a crucial role in the stages of differentiation and maintenance of insulin producing β-cells. It has been demonstrated that multipotent pancreatic progenitors adopt an acinar cell identity in the absence of Nkx6.1 activity [14]. Sander *et al.* [4] showed that the disruption of the Nkx6.1 gene causes severe effects in β-cell differentiation in mice and in Nkx6.1 null mutants have normal numbers of insulin expressing cells through e12, after this day new β-cell formation is blocked.

During pancreatic development, Nkx6.1 is expressed in a subset of Ngn3^+^ (marker transcription factor of all endocrine precursor cells populations) cells, but the expression in these cells is not significant enough to confer β-cell identity to Ngn3^+^ progenitors. β-cell formation requires Nkx6.1 activity in the Pdx1^+^ domain of progenitor cells and mediates beta cell maturation when is expressed in this domain [15]. This finding supports the results of Ahlgren *et al.* [9] who showed that β-cells lose Nkx6.1 expression upon Pdx1 deletion in these cells. Iype *et al.* [3] have also shown that Pdx1 can activate Nkx6.1 transcription during β-cell differentiation. It has been demonstrated that Pdx-1 maintains Nkx6.1 expression in mature β cells [16]. These findings and the observation that Nkx6.1 maintains Pdx1 expression during β-cell differentiation suggest the possibility that Pdx1 and Nkx6.1 cooperate to establish and maintain β-cell identity [10]. Schaffer *et al.* [10] reported that Nkx6.1 deficient β-cells adopted delta cell identity suggesting that Nkx6.1 is necessary for maintaining β-cell identity. These findings demonstrate that Nkx6.1 participates at the differentiation stage and functions in the preservation of β-pancreatic cells.

In mature β-cells, it has been demonstrated that Nkx6.1 is necessary for maintaining the level of insulin transcription, suppressing glucagon expression, for the normal physiologic release of insulin in response to elevated extracellular glucose [17]. It has been shown that the homeodomain Nkx6.1 preferentially binds to 5'-TAATTA-3' containing sequence *in vitro* which are similar to sequences (with TAAT) found in the insulin promoter region [18]. Taylor *et al.* [19] reported that the C terminus of Nkx6.1 (conserved across species) enhances DNA binding selectivity to the insulin promoter region (in which two of these sequences are localized), suggesting this may also improve *in vivo*.

Our model of partial pancreatectomy is a procedure known to stimulate the regenerative growth of β-cells in normal adult rodents in a process recapitulating embryonic development. Studies produced by Peshvaria *et al.* [20] in partially pancreatectomized mice showed an increase of β-cell mass and β-cell clusters. Li *et al.* [21] provide evidence that mature pancreatic duct cells can recapitulate the embryonic differentiation program during the regeneration after rat pancreatectomy. Through RT-PCR studies, an enhanced expression of numerous pancreatic progenitor markers have been shown, including Pdx-1 and Nkx6.1, in proliferation ductules compared with common pancreatic duct of both, unoperated or sham operated rats. According to these findings, in our study the partially pancreatectomized untreated group showed an increase in Nk6.1 gene expression, insulin gene expression, β-cell neogenesis in comparison to control group. Nevertheless, this response was not able to increase the serum insulin level to normalize the serum glucose level. In contrast, silymarin treatment produced an increase of these parameters that normalized serum glucose levels.

The increase in Nkx6.1 gene expression induced by silymarin treatment of partially pancreatectomized animals may be related to the increase of Pdx-1 expression observed in partially pancreatectomized animals treated with silymarin [22]. As was demonstrated by Wang *et al.* [16], Pdx-1 maintains Nkx6.1 expression in mature β-cell. In addition, the flavonoid silymarin could induce a direct action on Nkx6.1 gene promoters. Some reports have also pointed out the flavonoids activation of gene promoter regions of proteins [23,24]. Schisler *et al.* [25] demonstrated that Nkx6.1 overexpression in rat islets cause an increase of them and stimulation of β-cell replication with retention of function, whereas in human islets it caused an increase in the level of [_3_H] thymidine incorporation that was twice the control level, along with complete retention of glucose-stimulated insulin secretion and they conclude that Nkx6.1 is among the very rare factors capable of stimulating β-cell replication with retention or enhancement of function. According to this finding, the increase of insulin expression and the neogenesis of β-cells caused by silymarin treatment in pancreatectomized animals may be induced by the increment of Nkx6.1 expression observed in this group, thereby producing an increase in serum insulin and the normalization of serum glucose. However, we observed a decrease in Nkx6.1 expression (days 42 and 63) (Figure 1) after silymarin treatment and it would be expect a maintained levels of this transcription factor expression. Many of the transcription factors genes involved in the β-cell differentiation are subject to either positive or negative transcriptional feedback by their respective encoded proteins [26,27,28,29]. Such feedback mechanisms may allow for either the rapid enhancement or attenuation of transcription in specific cell types, thereby permitting efficient spatial and temporal control of gene expression. It has been reported that Nkx6.1 exerts its regulatory activity by binding preferentially to TAAT-containing DNA sequences within promoters [18,30]. This DNA binding activity is uniquely modulated by its acidic COOH-terminal domain, which is known to lower the intrinsic affinity of its homeodomain for DNA [18]. Upon binding to DNA, Nkx6.1 acts as a potent repressor of gene transcription. This repression activity is mapped to the—NH_2_ terminal domain of the protein. Although Nkx6.1 is a potent transcription repressor, it has been proposed that Nkx6.1 contains a negatively charged domain in the COOH terminus; in some cases such domains have shown to activate transcription through the recruitment of basal transcriptional machinery [31,32]. Iype *et al.* [3] showed that this activation is transcriptional in nature and is indeed mediated by a discrete, acidic sequence within the COOH-terminal domain. This activation requires the interaction of Nkx6.1 with a specific A/T-containing sequence within a β-cell specific enhancer element in the Nkx6.1 gene. The count of Nkx6.1 labelled cells (Figure 4) shows that silymarin treatment produced a significant increase of these cells at any time of treatment in comparison with untreated pancreatectomized cells. There was no significant difference between the groups treated with silymarin, and there was a very similar number of labeled cells at 21 and 63 days, which may indicate that there is no apoptosis process at 63 days. The expression of this transcription factor decreased at this time which may be due to an autorregulation of Nkx6.1 gene expression by Nkx6.1 transcrition factor as feedback mechanism [3]. In support of Nkx6.1 cells labeled at 21 and 63 days, western blot analysis at these times (Figure 2) did not show any difference between them. According with these results, β-cell neogenesis results (Figure 5) indicate a sustained neogenesis and did not present differences at 21 and 63 days. This may suggest that there is no apoptosis process. Our results suggest that silymarin may induce a functional pancreatic regeneration that may ameliorate the reduction in β-cells observed in both types of diabetes mellitus. Recently, it has been shown that in diabetic type 2 db/db mice, and in pancreatic biopsies of type 2 diabetic humans there was a reduction in Nkx6.1 which caused β cell failure. Transgenic expression of the glutathione peroxidase-1, (an antioxidant enzyme) in db/db islet β cells restored nuclear Nkx6.1, and β cell function *in vivo* [33]. In addition to our results, silymarin has been demonstrated to have antioxidant pancreatic properties such as an increasing in glutathione content and antioxidant enzymes activity in alloxan-induced diabetes mellitus in rats [34,35]. This effect may contribute to maintaining adequate Nk6.1 levels and to the improvement of β pancreatic cells functions in both types of diabetes mellitus.

## 3. Experimental

Silymarin and all reagents were of analytical grade and obtained from Sigma Chemical Co. (St. Louis, MO, USA) and Promega or from local suppliers (Merck and J.T. Baker, México City, Mexico). The experiments in this study were performed following the guidelines stated in “Principles of Laboratory Animal Care” (NIH publication #85-23, revised 1985) and Mexican regulations “Norma Oficial Mexicana NOM-062-ZOO-1999, Especificaciones técnicas para la producción, cuidado y uso de animales de laboratorio” (published on 6 December 1999). The studies were approved by the ethical committee of the Biological Sciences Division Council from our University.

### 3.1. Animal Treatments

Male Wistar rats (200–220 g of b. wt.) were obtained from our animal facility, fed with Purina standard chow, and maintained at 20–22 °C with 12-h light/dark cycles. The animals were divided into the following groups: (1) pancreatectomy group: 30 rats were anesthetized with ketamine (50 mg/Kg) and xylazine (5 mg/Kg). 60% of pancreatic tissue was removed by gentle rubbing using cotton applicators. These animals were divided into six groups for the following sacrifice times: 3, 7, 14, 21, 42 and 63 days. Body weight and serum glucose were measured weekly for each fasted rat; (2) pancreatectomy + silymarin: the same schedule was followed as with group 1. Pancreatectomized animals were administered with silymarin daily (200 mg/Kg b.w. p.o.) [36,37] *n* = 30; (3) control: these animals underwent a sham surgery and were sacrificed before treatments began (*n* = 6). Each week, animals were fasted for 7 h. Blood glucose was measured with an automatic glucometer (Abbot Diabetes Care Ltd., Oxon, UK) in tail vein and its weight was registered.

### 3.2. Blood and Tissue Collection

For sacrifice, animals were anesthetized with sodium pentobarbital (50 mg/Kg b.w., i.p.). Blood was obtained by cardiac puncture. Pancreas was extracted by dissecting the abdominal cavity and removing fat tissue.

### 3.3. RNA Isolation and RT–PCR Assay for Nkx6.1 and Insulin

RNA was isolated from a fragment of pancreatic tissue (50 mg) of each animal under different experimental conditions described above and was prepared with the column method according to the manufacture’s protocol Promega (Madison, WI, USA). Briefly, the tissue was homogenized in 1,000 µL of lysis buffer. 350 µL of dilution buffer was added to 175 µL of lysate. The sample was heated at 70 °C for 3 min and centrifuged for 10 min then 250 µL 95% ethanol was added to the cleared lysate and mixed. The lysate was transferred to column and centrifuged for 1 min, 600 µL of wash solution was added and centrifuged for 1 minute. 50 µL of DNase was applied to column and incubated for 15 min at room temperature. After this time, 200 µL of DNase stop solution was added and centrifuged for 1 min and washed two times. To eluate the RNA, 100 µL of nuclease free water was added to membrane. RT-PCR was performed with 2 μg of RNA for the target gene and the ribosomal constitutive gene for semi-quantitation. Oligonucleotide primers were designed based on literature sequences: 5'- GGG CTT GTT GTA ATCGTC GT -3' sequence for the sense (5') primer and the 5'- ACT TGG CAG GAC CAG AGA GA -3' for the antisense (3') primer for Nkx6.1. [25]. The 5'- CCA GTT GGT AGA GGG AGC AG- 3' sequence for the sense (5') primer and the 5'- CAC CTT TGT GGT CCT CAC CT -3' for the antisense (3') primer for insulin [38] and, 5'- GTA ACC CGT TGA ACC CCA TT-3' for the sense (5') and 5'- CAA TCC AAT CGG TAG TAG CG-3' for the antisense (3') primer for ribosomal 18 s mRNA amplification [39]. Amplification was initiated with 5 min at 94 °C, followed by 35 cycles of 95 °C, 60 °C and 72 °C, 1 min for each step, and reaction products were sequence-verified. Products were separated on a 2% agarose gel followed by ethidium bromide staining and densitometry analysis using a Kodak EDAS 290 (Kodak, Rochester, NY, USA).

### 3.4. Serum Insulin Levels

At the end of treatments, the serum insulin levels were measured for each animal with Insulin (Rat) Ultrasensitive ELISA (Salem, NH, USA) according to the manufacturer’s protocol.

### 3.5. β-Cell Neogenesis Assessment

Fragments of the pancreatic tail were fixed in 4% paraformaldehyde in PBS and embedded in paraffin. Five µm-thick sections were obtained, deparaffinized and a double label imunohistochemistry was carried out for β cell neogenesis assay. The primary and secondary antibodies of this study were obtained from (Zymed Labs, Inc., San Francisco, CA, USA). Prior to immunolabelling, deparaffinized pancreatic 5 µm-thick sections were immersed in 70% ethanol for 10 min and washed with PBS. 0.3% H_2_O_2_ for 10 min was added and incubated in 0.5% IgG-free albumin in phosphate buffered saline (PBS) for 30 min. For insulin immunoreaction, sections were labelled with mAbs anti-insulin (1:100) for 1 h at 30 °C. HRP-rabbit anti-guinea pig IgG (1:50) (peroxidase conjugated secondary antibody) was added for 2 h at room temperature. 600 µL DAB (diaminobenzidine)/H_2_O_2_ for 5 min, was used to visualize the reaction. Sections were rinsed with PBS. For CK-20, neogenesis marker [40] immunoreaction, these sections were treated with 0.3% H_2_O_2_ for 10 min and were immunolabelled with mAbs anti-CK20 (1:100) at room temperature for 2 h. HRP-goat anti-mouse IgG (1:50) was added for 90 min at room temperature. NiCl_2_ (1%) + CoCl_2_ (1%) mixture was added to DAB/ H_2_O_2_ solution for color development for eight min. Then sections were counterstained with hematoxylin. Double immunolabel was seen in the color black. Sections were analyzed with a 40× objective. The total number of cells and double immunolabelled cells were counted in five tissue areas with a Leica Application Suite program on a Leica DM 1000 microscope.

### 3.6. Pancreatic Tissue Nkx6.1 Immunoassay

A Nkx6.1 immunoassay was made for the first immunolabel as described above (2.5 section). mAbs anti-Nkx6.1 (1:100) was utilized as a primary antibody and HRP-goat anti-rabbit IgG (1:75) as a secondary antibody. Immunolabelled sections were analyzed with a DM-1000 Leica microscope. Immunolabelled cells were counted as percentage of total cells in each field with 5 fields of each animal pancreatic tissue at different times after pancreatectomy.

### 3.7. Western Blot Analysis

A fragment (40 mg) of pancreas was homogenized in 1.0 mL of an ice-cold isolation solution (250 mM sucrose, 10 mM triethanolamine, 1 mg/mL leupeptin, and 1 mg/mL phenylmethylsulfonyl fluoride, pH 7.6) at 15,000 rpm with 3 strokes for 15 s with a tissue homogenizer (IKA Works, Inc., Wilmington, NC, USA). After homogenization, total protein concentrations were measured by Bradford protein assay (Bio-Rad Laboratories Inc., Hercules, CA, USA). Samples were stabilized by adding 1 vol of 2X Laemmli sample buffer to 1 vol of sample and heating at 70 °C for 10 min, 100 micrograms of protein from each sample were loaded into individual lanes and electrophoresed on 12% polyacrylamide-SDS minigels using a Mini-PROTEAN Tetra Cell electrophoresis apparatus (Bio-Rad Laboratories Inc.). The proteins were then transferred electrophoretically to PVDF membrane (Immobilon-P, Millipore, Billerica, MA, USA). After blocking with 5% skim milk in PBS-T (80 mM Na_2_HPO_4_, 20 mM NaH_2_PO_4_, 100 mM NaCl, and 0.2% Tween-20, [pH 7.5]) for 1 h, the membranes were probed overnight at 4 °C with primary antibodies (1:500) (Zymed Labs, Inc.) and then incubated with secondary horseradish peroxidase conjugated antibody (1:10000) (Zymed Labs, Inc.). Sites of antigen-antibody reaction were viewed using enhanced chemiluminescence substrate (Immobilon Western) and analyzed in EDAS 290 (Kodak).

### 3.8. Statistical Methods

ANOVA followed by Tukey and Dunnet tests was used to compare experimental and control groups (SPSS, Chicago, IL, USA) with significance at *p* < 0.05.

## 4. Conclusions

Our results suggest that silymarin may induce an increase of β-cells neogenesis which could lessen the significant reduction of β-pancreatic cell mass in both types of diabetes mellitus, where in type 1 patients, there is an 80%–90% destruction at detection time and in type 2, there is a significant decrease in the number of these cells compared to healthy subjects [41].
